# The Effectiveness of Internet-Guided Self-help Interventions to Promote Physical Activity Among Individuals With Depression: Systematic Review

**DOI:** 10.2196/38049

**Published:** 2022-12-12

**Authors:** Yiling Tang, Madelaine Gierc, Raymond W Lam, Sam Liu, Guy Faulkner

**Affiliations:** 1 School of Kinesiology University of British Columbia Vancouver, BC Canada; 2 Department of Psychiatry, University of British Columbia Vancouver, BC Canada; 3 School of Exercise Science, Physical & Health Education, University of Victoria Vancouver, BC Canada

**Keywords:** physical activity, eHealth, mobile health, mHealth, depression, systematic review, internet, mobile phone

## Abstract

**Background:**

Depression is a prevalent and debilitating mental disorder and a leading cause of disability worldwide. Physical activity (PA) interventions have been shown to alleviate depressive symptoms. However, not all patients have access to PA programing tailored for depression. Internet-guided self-help (IGSH) interventions may be an effective option for increasing PA among people with depression who cannot or prefer not to access supervised exercise treatment.

**Objective:**

We aimed to evaluate the effectiveness of IGSH interventions in increasing PA and alleviating depressive symptoms in people with depression.

**Methods:**

A systematic literature search was conducted for randomized controlled trials and quasiexperimental studies using 9 electronic databases. The review was registered in PROSPERO (2020 CRD42020221713).

**Results:**

A total of 4 randomized controlled trials (430 participants) met the inclusion criteria. Of these, 3 were web-based and 1 was app-based. Three studies found IGSH interventions to have medium to large effects on decreasing depressive symptoms but not on increasing PA compared with waitlist or usual care. One study showed increased self-reported PA but no significant difference in depressive symptoms in the intervention group compared with the control group. Goal setting was the most common behavior change technique used in the interventions. Dropout rates within the intervention groups were relatively low (0%-19%).

**Conclusions:**

Our findings suggested that IGSH PA interventions are feasible and have the potential to reduce depressive symptoms in people with depression. More well-designed and tailored interventions with different combinations of behavior change techniques, particularly those targeting the emotion domain, are needed to assess the overall effectiveness and feasibility of using IGSH interventions to increase PA among people with depression.

**Trial Registration:**

PROSPERO CRD42020221713; https://tinyurl.com/ysaua5bu

## Introduction

### Background

Depression is a chronic mental health condition characterized by sadness; anhedonia; and secondary physical, cognitive, and emotional symptoms. People with depression often experience lower quality of life [1-3], increased risk of suicide [4,5], and increased risk of having comorbid chronic diseases (eg, diabetes, asthma, chronic lung disease, and coronary heart disease), which can result in premature death [6,7]. Historically, it was estimated that >300 million people have experienced depression worldwide [8], and most people have never been diagnosed [6,9,10]. Surveillance studies have observed a marked increase in the prevalence and severity of depressive symptoms since the beginning of the COVID-19 pandemic. For instance, Bueno-Notivol et al [11] observed that the prevalence of self-reported depression worldwide was 7 times higher in 2020 (25%) than in 2017 (3.44%). Similarly, a meta-analysis by Nochaiwong et al [12] estimated that more than a quarter of people globally (28%) self-reported depressive symptoms during the COVID-19 outbreak in 2020*.*

Physical activity (PA)—“any bodily movement produced by skeletal muscles that results in energy expenditure [13]”—has long been recognized as an important health-promoting behavior. In recent years, it has also been shown to be beneficial in the prevention and treatment of depressive disorders [14,15]. Exercise, a subset of PA that is typically planned and structured with the goal of increasing or maintaining fitness [13], is now recommended as a monotherapy for mild to moderate depression in Canada [16]. PA presents several advantages over conventional treatments for depression (ie, psychotherapy and antidepressant medication). Among them, PA has minimal negative side effects, is affordable, and is potentially more accessible [17]. Rebar and Taylor [18] suggested that PA could be a cost-effective method for treating depression worldwide. As an additional benefit, a large body of literature confirmed the positive side effects of engaging in regular PA, including heightened health-related quality of life (ie, physical and mental well-being) [19], chronic disease prevention (including obesity, type 2 diabetes, coronary heart diseases, and several cancers), and reduced risk of premature mortality [20,21].

Globally, a large minority (31.1%) of adults do not meet the minimum recommended levels of PA [22]. Data from the 2016 to 2017 Canadian Health Measures Survey suggested that only 16% of Canadian adults meet the current PA recommendation of 150 minutes of moderate to vigorous PA (MVPA) per week [23]. People with depression are more likely to experience lower PA levels than those without depression [24,25], in part because of symptoms such as pain and discomfort, insomnia, cognitive difficulties, fatigue, and anhedonia [17,25,26]. Tailored approaches to help people with depression that initiate and maintain PA are needed [27].

### Internet-Guided Self-help Interventions

Internet-guided self-help (IGSH) interventions, a form of eHealth intervention, could be 1 mechanism for supporting PA behavior changes among people with depression. eHealth interventions are defined as those that use information and communication technologies to enable health care, including supporting health behavior engagement [28,29]. As such, eHealth interventions are quite broad and include just-in-time adaptive interventions, wearable technology, telehealth, and social media. Research has demonstrated that most people with mental disorders have an interest in trying eHealth interventions (such as smartphone apps) to monitor and manage their health concerns [30,31].

IGSH interventions are characterized by web-based or app-based programs that are primarily self-guided. Some will offer limited support from a professional or paraprofessional [32]. Compared with synchronous eHealth interventions that likely require costlier direct consultation (eg, telehealth, live internet-based therapy, and live-streamed exercise sessions) [33], IGSH programs delivered via the web or mobile devices have the potential for broad reach and scalability at a relatively low cost. They also allow participants to access the intervention content at their own pace [34-36]. These features may be particularly beneficial for people with depression who experience symptoms, such as fatigue and disrupted sleep patterns. In addition, many people with depression experience stigma that negatively affects treatment-seeking [37] and have preferences for managing symptoms on their own [38]. Offering self-help interventions could be potentially useful in increasing help-seeking rates by mitigating stigma [39].

Systematic reviews and meta-analyses have found that eHealth interventions are effective in increasing short-term PA participation in nonclinical populations, including young people [40,41], adults [42], and older adults [43,44]. Other research on eHealth interventions suggests that interventions that adopt theory or incorporate evidence-based behavior change techniques (BCTs) are generally associated with greater effects and adherence [45,46]. A systematic review of meta-analyses on the effectiveness of self-help and internet-guided interventions has suggested that these programs are effective in treating depression [36,47]. In fact, computerized cognitive behavioral therapy (CBT) has been recommended as a treatment for subthreshold or mild to moderate depression in the United Kingdom [48]. Similarly, a systematic review by Andersson and Cuijpers [49] suggested that guided internet-based psychological interventions were more effective than unguided interventions for depression among adults.

Less is known about whether IGSH PA interventions for people with depression are effective at (1) increasing PA engagement and (2) reducing depressive symptoms. To date, 2 reviews have investigated eHealth PA interventions for individuals with mental illnesses [50,51], and 1 review [52] specifically examined web-based interventions. However, these reviews investigated eHealth interventions for mental illness generally, rather than IGSH programs for depression specifically. These are noteworthy distinctions, as the strongest research evidence of the benefits of PA-based treatments is for depression [16]; to our knowledge, no clinical guidelines exist for PA-based treatment for anxiety, schizophrenia, or other mental health conditions. In addition, the reviews included both experimental and observational studies [50]. Given the rapid pace of technological development and growing concerns of physical inactivity among people with depression, it is likely that this research field has expanded in recent years. There is a need for an updated systematic review of high-quality studies specific to depression. Thus, the primary objective of this systematic review was to assess the effectiveness of IGSH interventions in promoting PA and alleviating depressive symptoms in people with depression. The secondary objective of this study was to understand study characteristics, such as attrition rates and intervention design, to explore factors associated with successful interventions and areas for future growth.

## Methods

This systematic review was guided by the PRISMA (Preferred Reporting Items for Systematic Reviews and Meta-Analyses) approach [53,54]. The protocol was registered in PROSPERO (CRD42020221713). The PRISMA checklist is provided in Multimedia Appendix 1.

### Searches

A total of 2 rounds of search were conducted in December 2020 and November 2021. The search strategy was developed in consultation with a university rehabilitation sciences librarian. In all, 7 electronic databases were searched: MEDLINE (via Ovid), PsycINFO (EBSCOhost), CINAHL (EBSCOhost), Web of Science, Cochrane Central Register of Controlled Trials (CENTRAL; via Ovid), Embase (via Ovid), and SportDiscus (EBSCOhost). In addition, OpenGrey and ProQuest Dissertations were searched to identify gray literature that matched the inclusion criteria. Finally, reference lists of the included studies were searched to identify additional eligible studies.

The December 2020 search used a combination of controlled vocabulary (eg, Medical Subject Headings) and keywords related to “physical activity,” “depression,” and “eHealth.” For comprehensiveness, the search included keywords for all types of eHealth interventions. The search strategies for all databases are presented in Multimedia Appendix 2. The selected keywords were obtained from previous systematic reviews and protocols in relevant areas.

A revised search was conducted in November 2021, with two changes: (1) the results were limited to the period from December 1, 2020, to November 5, 2021, and (2) the search strategy was updated to better reflect the inclusion criteria and reduce irrelevant records informed by the experience of the December 2020 search. For example, the keywords “text messaging” and “video conferencing” were deleted. The updated search strategies for all included databases are presented in Multimedia Appendix 3.

### Eligibility Criteria

The study selection criteria were based on the Population, Intervention, Comparison, Outcome, Study (PICOS) design framework [55,56]. Studies that met the following criteria were included in this review.

#### Participants

Individuals with a clinical diagnosis of depression (eg, the Diagnostic and Statistical Manual of Mental Disorders criteria) or individuals with clinically significant depressive symptoms based on a validated self-report tool (eg, Patient Health Questionnaire 9-Item or Beck Depression Inventory-II [BDI-II] scores) were considered eligible for inclusion. Studies that used nonvalidated items (eg, “Are you depressed? Yes/No”) or those who did not use recognized diagnostic criteria were excluded. There were no restrictions based on age, gender, nationality, or ethnicity.

#### Interventions

Interventions were considered eligible if they were delivered via web-based platforms or mobile apps (eg, via smartphones and tablets) and included content designed to increase PA (eg, asynchronous PA programing, education, and PA goal setting). Studies were included even if promoting PA was the secondary objective, so long as changes in PA were measured and reported. By definition, interventions were primarily self-guided or automatic; however, interventions that offered degrees of human support (eg, providing personalized feedback via telephone or email) were permitted. In contrast, interventions primarily based on in-person support (eg, telephone counseling and SMS text messaging) were excluded. Similarly, live-streamed interventions (eg, live Zoom yoga classes) were excluded, as these are synchronous events organized in a live internet-based space. There were no restrictions based on the type of PA, PA intensity, bout length, program frequency or duration, or follow-up period.

#### Comparison

Any comparators were considered for inclusion. For instance, a PA intervention delivered in person, an alternative eHealth intervention, or a waitlist control group. No restrictions were placed on the nature of the comparison group.

#### Outcomes

The primary outcome was a change in PA levels. Both device-based (eg, accelerometer and pedometer) and self-report (eg, PA diary and questionnaires) measures were considered. Secondary outcomes included changes in the severity of depression, assessed by validated measures, and the acceptability of treatment assessed by (1) reported indicators of intervention engagement [57] and (2) dropout rates at postintervention. Engagement was defined as the extent to which the participants undertook the intervention. Dropout rate, also called attrition rate, was defined as the percentage of participants who were randomized to a group but failed to complete it.

#### Study Design

Randomized controlled trials (RCTs) and quasiexperimental studies with control groups and pre- and posttest measurements were considered. Studies were eligible if they were a published original manuscript or thesis or dissertation. Papers were excluded if they did not contain original findings (eg, editorials or reviews), if they were research protocols or proposals, or if they were conference abstracts.

#### Other

Papers were considered eligible for inclusion if they were published in English. There were no limitations on the date of publication.

### Study Selection

**Figure 1:**
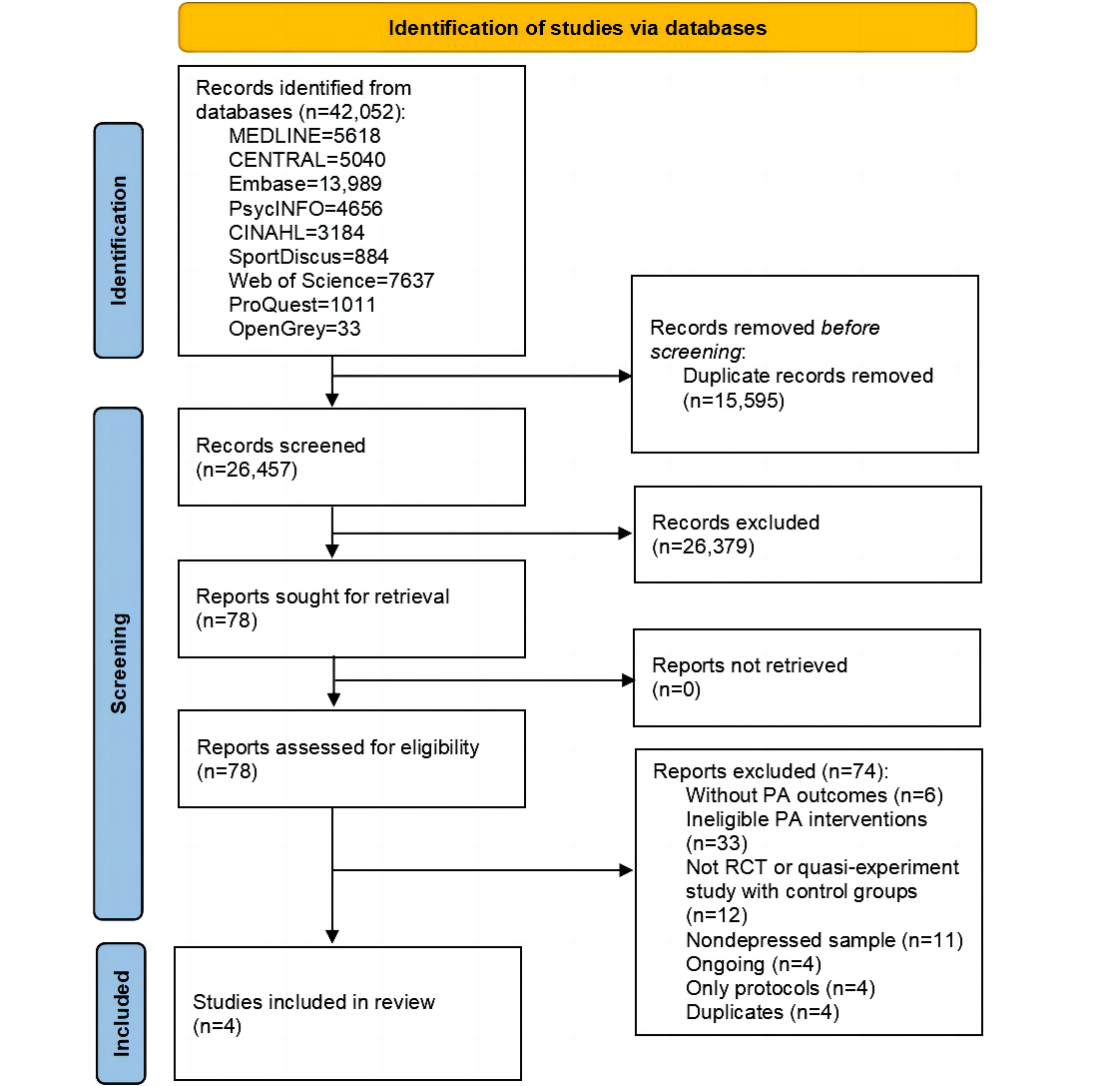
PRISMA (Preferred Reporting Items for Systematic Reviews and Meta-Analyses) flow diagram of the first round of searches. PA: physical activity; RCT: randomized controlled trial.

**Figure 2:**
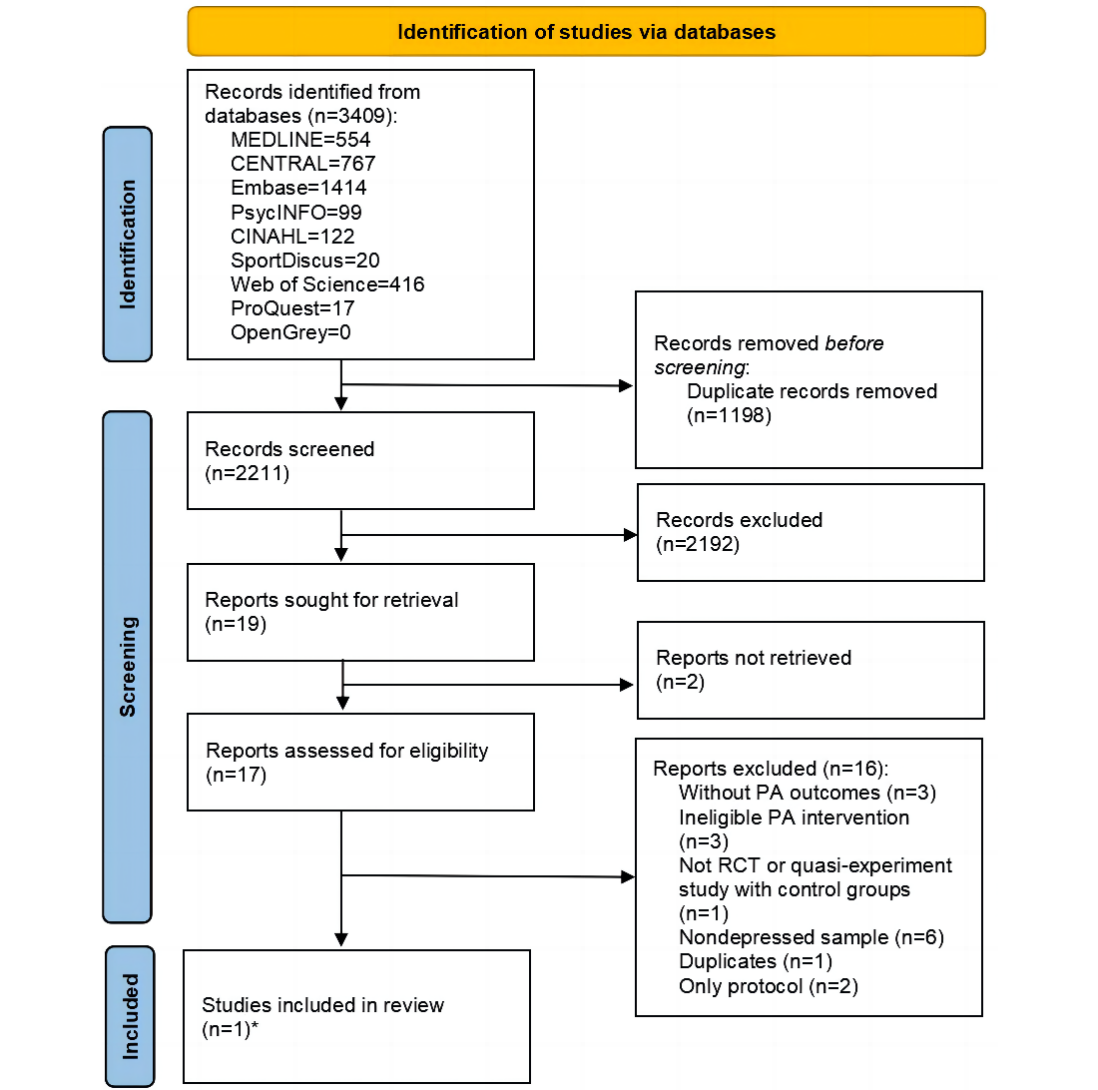
PRISMA (Preferred Reporting Items for Systematic Reviews and Meta-Analyses) flow diagram of the second round of searches. PA: physical activity; RCT: randomized controlled trial. *This study was excluded as being a duplicate of first round studies.

A total of 2 authors (first round: YT and JL; second round: YT and MG) independently screened the articles for inclusion using the agreed-upon study eligibility or ineligibility criteria. Screening occurred in 3 phases, facilitated by the Covidence systematic review software (Veritas Health Innovation). First, duplicate records were removed automatically using Covidence. Second, titles and abstracts were independently screened for relevance by 2 reviewers. In case of disagreement, the reviewers met to discuss and reach a consensus. Finally, full-text copies of the selected studies were screened (YT, JL, DD, and MG) to confirm their eligibility. Disagreements between the 2 researchers were resolved through discussion. A third reviewer (GF) was consulted if consensus could not be reached. Following selection, the reference list for each included study was searched to identify additional eligible studies. The PRISMA flow diagrams are presented in Figure 1 and Figure 2.

### Data Extraction

The following items were extracted by 1 author (YT) and reviewed for accuracy by another reviewer (first round: DD and second round: MG): (1) author and year; (2) study design; (3) country; (4) sample size; (5) participant characteristics, such as mean age and gender; (6) inclusion and exclusion criteria; (7) intervention details, including the level of contact; (8) comparator; (9) main outcomes (measurement tools); (10) additional outcomes (measurement tools); (11) main findings with regard to PA and depression symptomatology; (12) follow-up period; (13) BCTs; and (14) funding sources. A third author (GF) was consulted to resolve discrepancies.

Level of contact was categorized as high, moderate, and low, following procedures described by Ma et al [47]. If the support was directly provided by a qualified therapist (eg, exercise trainers providing personalized feedback), the level of contact was high. If the support was provided by members of the research team or trained students, it was considered to be moderate. If there was no direct contact with an interventionist (eg, only automated reminder emails were sent out), the contact was considered to be low.

### Risk of Bias

The Cochrane risk of bias tool (ROB 2; version 2) [58,59] was used to assess the risk of bias for both PA and depression outcomes. This tool includes 5 domains of risk of bias: randomization process, deviations from intended interventions, missing outcome data, measurement of the outcome, and selection of the reported result. The risk of bias for each outcome in each study was judged as high, some concerns, or low. Risk of bias was assessed by 2 independent reviewers (YT and MG). In case of disagreement, the reviewers met to discuss and reach a consensus. The remaining disagreements were resolved by consulting a third author (GF).

### Data Synthesis

Meta-analysis was not feasible because the included studies were too heterogeneous in their design and reporting of results. The study interventions and outcome characteristics were summarized using narrative synthesis and descriptive statistics. When the effect size was reported as Cohen *d* (standardized mean difference), it was interpreted as small (Cohen *d*=0.2), moderate (Cohen *d*=0.5), or large (Cohen *d*=0.8) [60].

### Confidence in the Cumulative Evidence

The Grading of Recommendation, Assessment, Development and Evaluation (GRADE) [61] criteria were used to rate the certainty of the cumulative evidence based on the risk of bias, imprecision, inconsistency, indirectness, and publication bias. The quality of evidence was categorized as high, moderate, low, or very low.

## Results

### Overview

A total of 45,461 records were identified: 42,052 in December 2020 and an additional 3409 in November 2021. After removing duplicates, 28,668 articles underwent title or abstract screening and 95 underwent full-text review. The first round of searches (December 2020) produced 4 articles. The second round of searches (November 2021) produced 1 additional article, which was subsequently deleted because it was a duplicate. A total of 4 eligible studies were included in this systematic review. The PRISMA flow diagrams are presented in Figure 1 and Figure 2.

### Characteristics of Included Trials

#### Study Design

Tables 1 and 2 summarize the characteristics of the 4 included studies. All 4 included studies were dual-arm RCTs. None of the studies used an active control group (eg, sham intervention) or treatment comparison group (eg, synchronous in-person therapy). Rather, the studies used either a waitlist or a treatment-as-usual group.

**Table 1:** Participant characteristics.

Study	Target population	Sample size	Age (years), mean (SD)	Sex (female or male)	Preexisting psychiatric treatment		
					Therapy	Medication	Combined
Guo et al [62], 2020	HIV and major depressive disorder	300; IG^a^: 150; CG^b^: 150	IG: 28 (5.8); CG: 28.6 (5.9)	IG: 8/142; CG: 15/135	0^c^	0^c^	0^c^
Haller et al [63], 2018	Adults (20-65 years old) with major depression	20; IG: 14; CG: 6	IG: 43 (14); CG: 51 (12)	IG: 10/4; CG: 3/3	IG: 2; CG: 0	IG: 7; CG: 3	IG: 3; CG: 3
Lambert et al [64], 2018	Adults with at least moderate depressive symptoms	62; IG: 32; CG: 30	IG: 39.3 (12.0); CG: 36.9 (12.6)	IG: 26/6; CG: 26/4	IG: 1; CG: 7	IG: 18; CG: 18	IG: N/A^d^; CG: N/A
Ström et al [65], 2013	Mild to moderate major depression diagnosis and sedentary lifestyle	48; IG: 24; CG: 24	IG: 48.8 (12.7); CG: 49.6 (8.7)	IG: 20/4; CG: 20/4	IG: 0; CG: 0	IG: 3; CG: 4	IG: N/A; CG: N/A

^a^IG: intervention group.

^b^CG: control group

^c^Potential participants excluded if they were currently on psychiatric treatment.

^d^N/A: not applicable.

**Table 2:** Characteristics of the included trials.

Study	Intervention	Duration of intervention (week)	Control	Attrition rate	Outcome measurements	Effect size
Guo et al [62], 2020	Cognitive behavioral stress management course+PA^a^ promotion via WeChat app	12	Waitlist control+usual care of HIV	8.7%; IG^b^: 11 cases; CG^c^: 15 cases	PA: METs^d^ calculated from Chinese version of the GPAQ^e^; depression: CES-D^f^ (main), PHQ-9^g^ (secondary)	PA outcome: nonsignificant between-group differences (3 mo MET: −1898 (−4285 to 489); *P*=.12); depressive outcome: between-group mean difference −5.77 (95% CI −7.82 to −3.71), Cohen *d*=0.66; *P*<.001
Haller et al [63], 2018	Web-based platform with weekly exercise schedules and motivational feedback, as well as an additional biweekly group training session	8	Treatment as usual	15%; IG: 3 cases; CG: 0 cases	PA: Baecke questionnaire; depression: QIDS-SR^h^ and QIDS-C^i^	Total HPA^j^ outcome: eta^2^=0.36; *P*=.007; Depressive outcome: after 6-12 days: nonsignificant between-group differences (QIDS-SR: *P*=.06; eta^²^=0.2) posttreatment: nonsignificant between-group differences
Lambert et al [64], 2018	Web-based modular-fashioned course with evidence-based treatment based on behavioral activation and PA promotion	8	Waitlist control+treatment as usual	19%; IG: 7 cases; CG: 5 cases	Device-based PA: min per week of objective MVPA^k^ in 10-min bouts; self-reported PA: IPAQ-SF^l^; depression: PHQ-8^m^	PA outcome: nonsignificant between-group differences—between-group adjusted mean difference: device-based PA: 16.4 (−43.7 to 76.5); self-reported PA: 0.2 (−8.7 to 9.2); depressive outcome: between-group adjusted mean difference −3.6 (95% CI −6.1 to −1.1); Cohen *d*=0.93
Ström et al [65], 2013	Web-based guided self-help PA program with 9 text modules, written feedback, and home assignments, from therapists	9	Waitlist control	0%; IG: 0 cases; CG: 0 cases	PA: IPAQ^n^; depression: BDI-II^o^ (main); and MADRS-S^p^ (secondary)	PA outcome: nonsignificant between-group differences (Cohen *d*=0.20); depressive outcome: between-group—BDI-II: Cohen *d*=0.67; MADRS-S: Cohen *d*=0.62

^a^PA: physical activity.

^b^IG: intervention group.

^c^CG: control group.

^d^METs: metabolic equivalents.

^e^GPAQ: Global Physical Activity Questionnaire.

^f^CES-D: Centre for Epidemiological Studies Depression.

^g^PHQ-9: Patient Health Questionnaire 9-item.

^h^QIDS-SR: Quick Inventory of Depressive Symptomatology—self-report.

^i^QIDS-C: Quick Inventory of Depressive Symptomatology—clinician rating.

^j^HPA: habitual physical activity.

^k^MVPA: moderate to vigorous physical activity.

^l^IPAQ-SF: International Physical Activity Questionnaire—Short Form.

^m^PHQ-8: Patient Health Questionnaire 8-item.

^n^IPAQ: International Physical Activity Questionnaire.

^o^BDI-Ⅱ: Beck Depression Inventory—second version.

^p^MADRS-S: Montgomery-Åsberg Depression Rating Scale: Self-rated version.

#### Sample Characteristics

The studies were conducted in 4 different countries: China [62], Germany [63], the United Kingdom [64], and Sweden [65]. Together, the 4 studies included 430 people with depression. The sample size varied from 20 [63] to 300 [62]. Guo et al [62] included adults with a clinical diagnosis of HIV, with the majority (92.3%) of participants being men. The remaining 3 (75%) studies included higher rates of female participation (65% [63] to 86.7% [64]). The mean age ranged from 28.3 (SD 5.8) [62] to 49.2 (SD 10.07) years [65]. The 3 (75%) studies [63-65] included participants receiving psychiatric treatment, including psychotherapy or medication. Although the studies varied in their reporting of sociodemographic characteristics, it was generally observed that the samples were well-educated (ie, the majority of participants had completed high school or more) and currently employed. In addition, the 3 (75%) studies [62,64,65] reported participants’ baseline PA levels, with different proportions of them being physically active (13% [64] to 60.5% [65]).

#### Measures

Of the 4 studies, 3 (75%) used self-report methods to measure PA: metabolic equivalents calculated from the Global Physical Activity Questionnaire [62], habitual PA scores calculated from the Baecke Physical Activity Questionnaire [63], and the International Physical Activity Questionnaire [65]. In contrast, Lambert et al [64] used a combination of self-report (International Physical Activity Questionnaire–Short Form) and device-based measures (accelerometry, with MVPA reported as minutes per week in 10-minute bouts).

#### Intervention Characteristics

Interventions typically provide discrete “modules” of information with distinct learning objectives. One study by Guo et al [62] delivered the intervention via a preexisting commercial app (WeChat). The other 3 (75%) trials used web-based PA interventions. The intervention length ranged from 8 weeks [63,64] to 3 months [62]. In addition, 2 (50%) studies [63,64] reported short-term postintervention effects, whereas Guo et al [62] and Ström et al [65] assessed longer-term maintenance (6- and 9-month and 6-month maintenance, respectively). All 4 studies incorporated weekly contact. Moreover, 2 (50%) studies [63,64] reported a moderate level of contact. Haller et al [63] and Ström et al [65] included a high level of contact with participants receiving feedback from a therapist via phone or an encrypted web-based platform, respectively. In addition, Haller et al [63] incorporated an optional biweekly face-to-face training session led by a sports therapist.

### Attrition and Engagement

Dropout rates were relatively low, ranging from 0% [65] to 19% [64] across the 4 studies (Table 2). In terms of intervention engagement, participants in the study by Guo et al [62] completed 55% of the program (ie, 9 content modules and 3 review modules). In the study by Haller et al [63], participants were recommended to complete a maximum of 3 endurance sessions and 2 strength training sessions per week for 8 weeks. Participants completed 84% (16 [IQR 9-19] of 19 [IQR 15-21]) of recommended endurance sessions and 90% (9 [IQR 4-12] of the 10 [IQR 8-13]) of recommended strength training sessions. In the study by Lambert et al [64], only 1 participant used all 13 modules: the introduction module, 8 weekly modules, 1 generic problem-solving module, and 3 unlockable modules. A total of 53% (17/32) of the participants completed at least the introduction module and the first 2 weekly modules, and 25% (8/32) participants completed at least 4 weekly modules. In the study Ström et al [65], 58% (14/24) of the intervention participants completed all the 9 modules.

### Effectiveness of Interventions

All 4 studies [62-65] reported a change in depressive symptoms as the main outcome and change in PA as a secondary outcome. All studies observed a positive effect of the intervention on depression severity between baseline and posttreatment, 3 (75%) of which [62,64,65] reported significant and moderate or large between-group differences.

The app-based intervention by Guo et al [62] found a moderate to large effect on depressive symptoms in the intervention group versus the waitlist control group (mean difference −5.77; 95% CI −7.8 to −3.71; Cohen *d*=0.66; *P<*.001), which was sustained at 6 months (Cohen *d*=0.63; *P<*.001) and 9 months (Cohen *d*=0.51; *P<*.001) after intervention. However, there were no significant changes in PA levels (metabolic equivalents) from baseline to follow-up in either group.

Haller et al [63], Lambert et al [64], and Ström et al [65] all examined web-based PA interventions. Similar to Guo et al [62], Haller et al [63] reported significantly reduced depressive symptoms on the Quick Inventory of Depressive Symptomatology Clinician Rating (*P=*.02) and Self-Report Quick Inventory of Depressive Symptomatology (*P=*.001) from before to after the intervention. However, there were no significant differences between the intervention and control groups in reducing depressive symptoms after 6 to 12 days (η^2^=0.2; *P=*.06) and after treatment. In terms of PA improvement, Haller et al [63] reported significantly increased total habitual PA (η^2^=0.36; *P=*.007) in the intervention group after the 8-week intervention. In the study by Ström et al [65], the web-based intervention was effective in reducing depressive symptoms, reflected both in Montgomery-Åsberg Depression Rating Scale: Self-rated version and BDI-II. For example, the BDI-II results showed a moderate between-group effect size (Cohen *d*=0.67; 95% CI 0.09-1.25). Ström et al [65] also reported increased PA levels in both the intervention group and the control group. However, this change did not significantly differ between groups. In Lambert et al [64], the results showed greater changes in depressive symptoms (Patient Health Questionnaire 8-item scores: adjusted mean difference −3.6; 95% CI −6.1 to −1.1) at 2 months in the intervention group. The intervention group also reported a higher median of minutes of device-measured MVPA in 10-minute bouts (97.6 [IQR 49.7-166.3]) than the control group (13.0 [IQR 0.0-131.4]), although this difference was not statistically significant.

### Theory and BCTs

A large body of evidence illustrates that interventions are more effective when they are informed by theory and integrated with evidence-based BCTs. The studies by Lambert et al [64,66] were the only study that explicitly describes the adoption of a behavior change or knowledge translation framework, the Centre for eHealth Research and Disease Management road map, to guide intervention design and evaluation.

Three (75%) studies were informed by a therapeutic approach: Guo et al [62] adopted cognitive behavioral and stress management principles; Lambert et al [64] was informed by behavior activation; and Ström et al [65] incorporated aspects of acceptance and commitment therapy (ACT) and motivational interviewing. With regard to behavior change theories, Lambert et al [64] applied Self-Determination Theory (SDT) to behavioral activation as the underlying theory of PA behavior change and Ström et al [65] was informed by the SDT and the Transtheoretical (Stages of Change) Model. The remaining studies [62,63] described an atheoretical or eclectic approach that integrated a few common BCTs for PA promotion.

BCTs for increasing PA were coded according to Michie et al [67] BCT Taxonomy. In the intervention groups, “goal setting (behavior)” (n=4), “problem solving” (n=3), “review behavior goals” (n=3), “feedback on behavior” (n=3), “self-monitoring of behavior” (n=3), and “information about health consequences” (n=3) were the 6 most commonly used BCTs. The highest number of BCTs were identified in both Ström et al [65] (n=15) and Lambert et al [64] (n=15). Table 3 provides an overview of BCT use across the studies.

**Table 3:** Intervention behavior change techniques for physical activity promotion (N=4).

BCT^a^	Guo et al [62], 2020	Haller et al [63], 2018	Lambert et al [64], 2018	Ström et al [65], 2013	Total (n/N)
Number of BCTs, n	7	8	15	15	—^b^
1.1 Goal setting (behavior)	✓^c^	✓	✓	✓	4/4
1.2 Problem solving	✓	—	✓	✓	3/4
1.4 Action planning	—	—	✓	✓	2/4
1.5 Review behavior goals	—	✓	✓	✓	3/4
1.7 Review outcome goals	—	—	—	✓	1/4
1.8 Behavior contract	—	✓	—	—	1/4
2.2 Feedback on behavior	✓	✓	—	✓	3/4
2.3 Self-monitoring of behavior	✓	—	✓	✓	3/4
2.6 Biofeedback	—	✓	—	—	1/4
3.3 Social support (emotional)	—	✓	—	—	1/4
4.1 Instructions on how to perform the behavior	✓	✓	✓	—	3/4
4.4 Behavior experiments	—	—	✓	—	1/4
5.1 Information about health consequences	✓	—	✓	✓	3/4
5.4 Monitoring of emotional consequences	—	—	✓	—	1/4
5.6 Information about emotional consequences	—	—	✓	✓	2/4
6.1 Demonstration of the behavior	—	✓	✓	—	2/4
7.1 Prompts or cues	—	—	✓	—	1/4
8.3 Habit formation	—	—	—	✓	1/4
8.7 Graded tasks	—	—	✓	—	1/4
9.1 Credible source	—	—	✓	—	1/4
9.2 Pros and cons	—	—	—	✓	1/4
9.3 Comparative imagining of future outcomes	—	—	—	✓	1/4
10.4 Social reward	—	—	✓	—	1/4
10.9 Self-reward	—	—	—	✓	1/4
11.2 Reduce negative emotions	✓	—	—	—	1/4
15.3 Focus on past success	—	—	—	✓	1/4
16.2 Imaginary reward	—	—	—	✓	1/4

^a^BCT: behavior change technique.

^b^Not available.

^c^Indicates that a behavior change technique was used.

### Risk of Bias Within Studies

The ROB 2 [58,59] tool was used to assess the risk of bias for each outcome (PA and depression) of each included RCT (Figure 2). Overall, the “randomization process” was properly used in all studies. In addition, there were no concerns related to “deviations from the intended interventions” and “selection of the reported result.” However, in terms of PA, 2 (50%) studies [63,64] were judged as having a high risk of overall bias owing to “missing outcome data.” Three (75%) studies [62,63,65] showed some concerns owing to the subjective measurement of PA outcomes. In terms of depression, 3 (75%) studies [62-64] presented concerns owing to missing outcome data or outcome measurements.

### Quality of Cumulative Evidence

The Grading of Recommendation, Assessment, Development and Evaluation assessments are presented in Table 4. The 4 selected RCTs started as high-quality evidence but were subsequently rated down owing to inconsistency and imprecision.

**Table 4:** Quality of evidence of outcomes of interest.

Outcomes	Risk of bias	Publication bias	Inconsistency	Indirectness	Imprecision	Quality of evidence
Physical activity	Some concerns to high^a^	Not suspected	Serious	Not serious	Serious	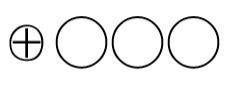 Very low
Depression	Low to some concerns^a^	Not suspected	Not serious	Not serious	Serious	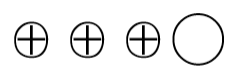 Moderate

^a^Detailed information is presented in Figure 3.

**Figure 3:**
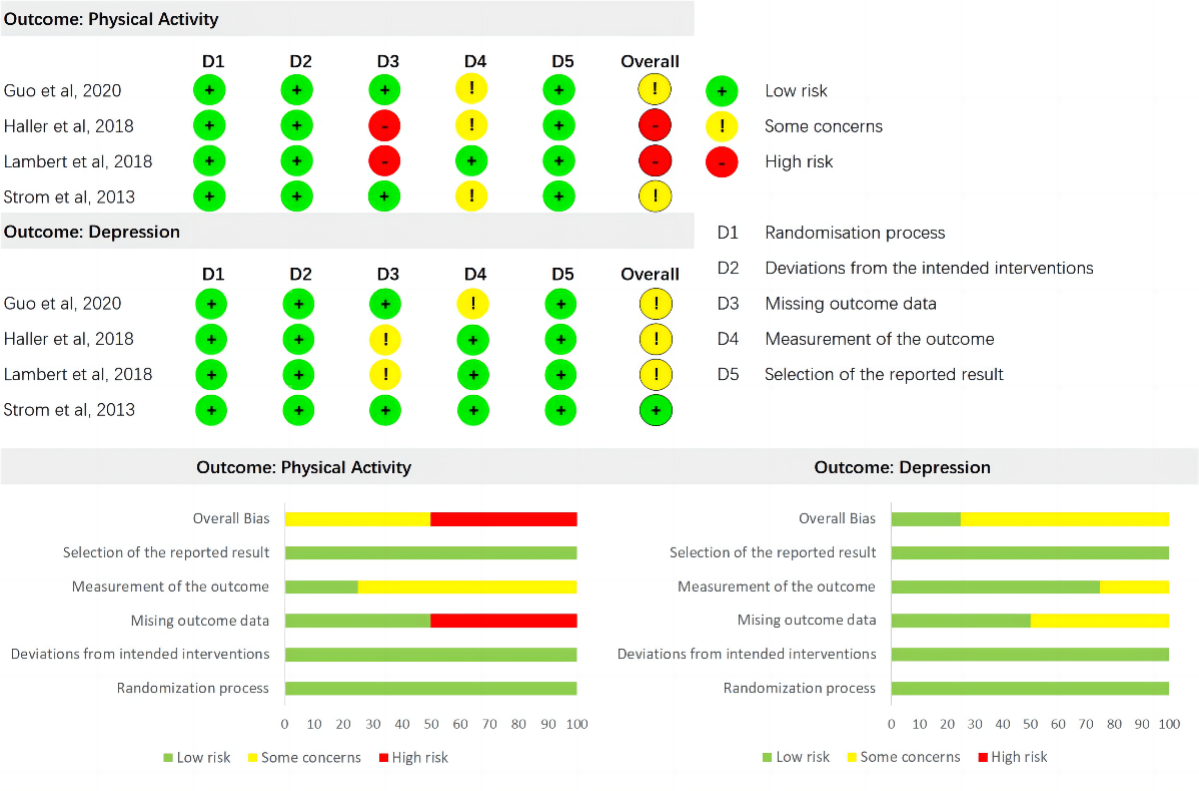
Risk of bias assessments of physical activity and depression of included studies.

## Discussion

### Principal Findings

To our knowledge, this is the first systematic review of IGSH interventions to increase PA in people with depression. A total of 4 studies met our inclusion criteria: 3 (75%) web-based RCTs and 1 (25%) app-based RCT. The 3 (75%) studies [62,64,65] reported that PA levels increased at postintervention in both treatment and control groups. Overall, between-group differences in PA were small and generally not significant. In contrast, 3 (75%) studies found significant medium to large reductions in depressive symptoms; the final study reported a significant reduction in both the intervention and control groups. Collectively, these results suggested that IGSH interventions may be helpful for managing clinical depression.

Why did these interventions fail to increase PA levels? Although explanations may vary across studies, several common factors were noted. In 2 studies [62,64], PA was presented as part of a larger depression management strategy, in conjunction with other components. For example, the Run4Love intervention by Guo et al [62] featured sessions on cognitive behavioral and stress management strategies. As PA was not the primary focus of these interventions, the nonsignificant results were not altogether surprising. More-intensive interventions, that is, those with a central focus on PA behavior change, may be required to produce clinically meaningful changes in PA behavior. From a methodological perspective, the 3 (75%) studies had small sample sizes, which is associated with an increased risk of type 2 error [68].

An additional explanation for the nonsignificant differences in PA is low engagement. Low engagement has previously been identified as a major challenge to eHealth interventions and is likely to negatively impact the effectiveness of interventions [69-71]. In our review, 3 of the 4 (75%) studies were marked by low participant engagement despite different tactics to enhance adherence (eg, reminders via phone calls or emails and tailored feedback). In addition, Lambert et al [64] included some level of tailored content by incorporating graded tasks, where participants could select their preferred physical activities (easier to harder) for the following week from week 3 onward. However, 47% (15/32) of the participants did not reach week 3. The only study with a positive effect on PA change (Haller et al [63]) reported a remarkably higher adherence rate, with participants completing 84% of the recommended endurance sessions and 90% of recommended strength sessions. This study not only tailored the exercise duration or intensity for participants from the beginning of the intervention but also provided weekly personalized feedback. This indicated that personalized IGSH interventions could potentially be more effective than standardized interventions in increasing PA—something that is well-established in the broader literature [72]. Similarly, the importance of personalized components in engagement with technology-based interventions has been emphasized by both people with depression and practitioners [73-75]. Additional supportive strategies, such as providing weekly individualized feedback to enhance adherence, should be incorporated throughout the intervention. Future IGSH interventions should consider combining tailored content based on participants’ preferences and conditions (physical or mental) and weekly personalized feedback for potentially improving engagement with the intervention content.

In addition, the types of measurements used may have affected the results. Only Lambert et al [64] incorporated both subjective and device-based PA measurements. Even then, less than half of the participants in both the intervention and control groups provided valid device-based PA data, which may have significantly affected PA outcomes. The other 3 (75%) studies [62,63,65] relied solely on self-reported PA measures, which were subject to recall bias. It is worth noting that none of these self-reported PA measures were developed specifically for people with mental health disorders and may fail to accurately assess PA in the selected studies [76]. Moreover, 3 (75%) studies [62,64,65] incorporated a “self-monitoring of behavior” BCT component. For example, Ström et al [65] provided the intervention group with a pedometer. Consequently, we would expect the treatment and control groups to differ in their awareness and ability to recall PA. Finally, approximately half of the participants were physically active before enrolling in the study [62,64]. In short, PA promotion effects may be influenced by ceiling effects.

### Depressive Symptoms

In contrast to PA outcomes, more consistent evidence was found for depressive symptom reduction. Three of the included studies [62,64,65] reported a medium to large effect on decreasing depression symptoms relative to controls. The final study [63] reported a significant reduction in both the intervention and control groups. These results were consistent with a previous meta-analysis that found IGSH interventions to be effective in reducing depression among college students [47]. As PA is generally unaffected by these interventions, the improvements observed across studies are likely attributable to the various therapeutic components used. Within the field of clinical psychology, evidence-based therapies are regularly informed by theory; for example, CBT, behavioral activation, ACT, and interpersonal therapy all possess distinct theoretical foundations that uniquely guide case conceptualization and treatment activities. The 3 studies incorporated ≥1 therapeutic modalities: cognitive behavioral and stress management approaches [62], behavioral activation [64], and ACT and motivational interviewing [65]. This is in line with an earlier study suggesting that therapist-guided web-based CBT has a large effect on depression outcomes [77], and other studies suggesting behavioral activation strategies, delivered either in person or over the internet, can be as effective as CBT for depression management [78-80].

One of the 4 studies [63] did not observe a significant between-group decrease in depressive symptoms, although early antidepressive findings (6-12 days) were noted on the threshold of significance (*P=*.06). There were 2 possible explanations. First, the sample size was small (n=20) and the groups were unequal (intervention group=14; control group=6). Therefore, it is likely that this study was underpowered [81]. Second, the authors observed that 1 of the 6 participants in the control group reported a full remission from severe depression after treatment. Although not impossible, this is an unusual occurrence and likely resulted in an inflated index of depressive symptom reduction in the control group. Therefore, the between-group difference was considered less reliable. In contrast, the significant and meaningful reduction in depression in the IG group was believed to be attributed to PA participation. In short, all studies reported that IGSH interventions are feasible and effective in treating depression.

### Intervention Characteristics and BCTs

In general, research suggests that theory-based interventions are more effective than interventions without theory [45,46,82]. In addition to providing a framework for selecting evidence-based BCTs, theories also aid in understanding factors that mediate behavior changes and the reasons for intervention success or failure [83,84]. Only 1 study in this review explicitly identified the empirical or theoretical basis for BCT selection for PA promotion. Specifically, Lambert et al [64,66] reported that SDT was adopted to inform their eMotion intervention. Similarly, Ström et al [65] were inspired by an earlier study that used SDT as the theoretical framework. Apart from theories, the importance of intervention development frameworks for guiding the development process of behavior change interventions has also been highlighted to reduce the risk of research waste and increase the effectiveness and sustainability of interventions [85,86]. Only Lambert et al [64,66] identified a framework: in their case, the Center for eHealth Research and Disease Management road map.

In contrast to theory, all 4 interventions used multiple BCTs. The most common BCTs identified were goal setting, problem solving, feedback, reviewing behavioral goals, providing information about health consequences, and instructions on how to perform the behavior. As noted by Bohlen et al [82], these BCTs are frequently used in interventions for the general population. Within the literature, there is growing interest in how to optimally combine BCTs to support behavior change, with several studies suggesting that BCT combinations might differ across populations and behavioral targets [87-90]. A previous factorial trial of an internet-based intervention [91] found that combining action planning, coping planning, and self-monitoring induced and amplifies the effect of increasing MVPA in healthy adults. Interestingly, both Lambert et al [64] and Ström et al [65], who included this combination of BCTs, reported no change in PA behavior compared with the control groups. Although methodological limitations must be emphasized, this observed inconsistency raised the question of tailoring BCTs to the population. We cannot assume that strategies that are effective for the general population are a good fit for people with depression. Rather, different or additional BCTs may be required to support PA promotion.

A scoping review of the barriers to and facilitators of exercise in people with depression suggested that interventions should incorporate BCTs that target the emotion domain (eg, to address low mood) [92]. Cane et al [93] recommended 4 BCTs for the emotion domain: “social support (emotional),” “information about emotional consequences,” “self-assessment of affective consequences,” and “reduce negative emotions.” In our review, all 4 studies [62-65] addressed aspects of emotion (eg, both Lambert et al [64] and Ström et al [65] touched upon the emotional consequences of PA), although none of the studies used all 4 BCTs to target this domain. The lack of population-tailored BCTs might partly explain the inconsistent results regarding PA outcomes. Future IGSH interventions for PA behavior change among people with depression should test different combinations of BCTs and consider integrating more BCTs that focus on the emotion domain.

### Attrition and Engagement

The included IGSH interventions showed relatively low dropout rates (0%-19%). In contrast, Meyerowitz-Katz et al [94] reported a dropout rate of 40% (95% CI 16%-63%) among RCTs on mobile health interventions. Josephine et al [95] also found a mean intervention dropout rate of 37% within internet-based and mobile-based interventions for people with depression. There have been some studies suggesting that higher levels of therapy contact have a positive impact on acceptance of the intervention [96,97]. All the included studies incorporated at least a moderate level of participant contact, including weekly contact with study-affiliated personnel. Three studies [62,63,65] provided individualized support; these reported lower attrition and higher study engagement compared with the study by Lambert et al [64], who provided weekly automated reminder emails. This is consistent with previous studies that suggested regular motivational feedback may enhance the adherence to internet-based intervention [98,99]. Similarly, our findings suggest that regular individualized feedback may contribute to reduced dropout rates. Further research is needed to explore the effects of different levels of interventionist contact on IGSH interventions.

### Strengths and Limitations

This review provides an updated summary of IGSH interventions for PA promotion in people with depression. Two rounds of searches were conducted to ensure inclusion of all available evidence. A notable strength of this review was the application of the BCT taxonomy to identify potentially useful ingredients for informing future intervention development. We addressed the gaps observed in previous reviews by including only RCTs and specifically focusing on people with depression.

Similar to all studies, this review was not without limitations. First, both the limited number of studies in this review and their heterogeneity in measurement precluded the meta-analysis and limited our ability to draw clear conclusions. Encouragingly, this is a rapidly evolving field, and several promising protocols were identified during article screening (eg, study by Sylvia et al [100]). We are hopeful that more evidence will become available in the next several years. Second, none of the studies had a primary outcome of PA, and because of the measures used, there is a very low level of certainty of PA evidence included. Third, only studies published in English were searched and screened. Fourth, this study used an a priori definition of IGSH interventions, which conceptualized these interventions as technology-facilitated and primarily self-guided, with the option of in-person support. Although having a clear definition is a strength, we did not explicitly designate the degree of in-person support that would render the studies ineligible. Study selection was determined by researchers through a process of discussion. As such, some studies were excluded from this study, which other research groups may have considered eligible or ineligible. Finally, we coded the BCTs using a dichotomous system (ie, yes or no). This review was unable to speak to the quality and rigor of BCT administration within the included studies.

### Future Directions

Despite increasing interest in eHealth and IGSH interventions, this review identified only 4 studies that (1) included a PA intervention component, (2) assessed changes in PA, and (3) specifically targeted people with depression. However, there were notable gaps in the research design. None of the 4 identified studies identified PA behavior change as their primary objective; interventions contained relatively little content focused on PA promotion; studies generally featured small samples; and half of the included studies did not incorporate behavior change theory for BCT selection or follow a systematic framework of behavioral intervention development. In contrast, not only is PA now recommended as a first-line monotherapy for mild to moderate depression but also there is a large and robust body of evidence regarding the different theories, BCT, and characteristics that can support successful eHealth or IGSH intervention. In summary, there appears to be a large gap between general research and its specific application in IGSH interventions to promote PA in people with depression.

Therefore, there is a great need for high-quality and thoughtful research on IGSH interventions for people with depression. On the basis of the results of this review, future research should be characterized by the following: (1) a specific focus on PA promotion within people with depression, including diverse populations of people with depression (eg, older adults, racialized communities, and individuals in rural or remote communities); (2) a priori consideration of theory, including using theory to guide BCT selection; (3) a rigorous development process following a systematic intervention development framework; (4) defining specific intervention targets (eg, meeting Canadian Network for Mood and Anxiety Treatments Guidelines [16] of 3×30-minute bouts of MVPA per week); (5) exploration of the mediators and moderators of behavior change, as defined by theory; and (6) using validated tools to assess before and after changes in depressive symptoms and PA, with inclusion of objective measures when possible. Further questions for examination included exploring the optimum amount and modality of guided support, personalization and tailoring considerations, PA programing (eg, frequency, intensity, type, and time), the moderating effect of patient characteristics (eg, baseline fitness and symptom severity), and knowledge translation and intervention scaling.

### Conclusions

An emerging body of evidence suggests that IGSH PA interventions are feasible and have the potential to reduce depressive symptoms in people with depression. More well-designed and tailored interventions are needed to assess the overall efficacy and feasibility of using IGSH interventions to help people with depression increase PA. Future research on such interventions should be theoretically informed in its development and implementation and test different combinations of BCTs, particularly those targeting the emotion domain, to verify their efficacy in increasing PA among people with depression.

## Appendix

Multimedia Appendix 1 PRISMA (Preferred Reporting Items for Systematic Reviews and Meta-Analyses) 2020 checklist.

Multimedia Appendix 2 Search strategies for the first round of searches.

Multimedia Appendix 3 Search strategies for the second round of searches.

## Abbreviations

ACT: acceptance and commitment therapy
BCT: behavior change technique
BDI-II: Beck Depression Inventory-second version
CBT: cognitive behavioral therapy
IGSH: internet-guided self-help
MVPA: moderate to vigorous physical activity
PA: physical activity
PICOS: Population, Intervention, Comparison, Outcome, Study
PRISMA: Preferred Reporting Items for Systematic Reviews and Meta-Analyses
RCT: randomized controlled trial
SDT: Self-Determination Theory
