# Supplementary material for: The Effectiveness of Internet-Guided Self-help Interventions to Promote Physical Activity Among Individuals With Depression: Systematic Review
Source: JMIR Ment Health. 2022 Dec 12;9(12):e38049. doi: 10.2196/38049 (PMC9793299; doi:10.2196/38049)
Supplement: Multimedia Appendix 2 [file mental_v9i12e38049_app2.docx]

Multimedia Appendix 2. Search strategies for the first round of searches

Search Strategy for MEDLINE (Ovid)

| Search number | Query |
| --- | --- |
| 1 | depression/ or exp Depressive Disorder/ or Dysthymic Disorder/ or Mood Disorders/ or Affective Symptoms/ or Adjustment Disorders/ |
| 2 | (Depress* or melancholia* or Dysthymi* or Mood disorder* or affective disorder* or Affective symptom* or Adjustment disorder* or Reactive disorder*).mp. [mp=title, abstract, heading word, drug trade name, original title, device manufacturer, drug manufacturer, device trade name, keyword, floating subheading word, candidate term word] |
| 3 | 1 or 2 |
| 4 | telemedicine/ or telerehabilitation/ or exp Internet/ or exp Videoconferencing/ or exp Cell Phone/ or wearable electronic devices/ or fitness trackers/ |
| 5 | (Mobile health or Telehealth or Telemedicine or Telerehabilitation or E-rehab* or Ehealth or Mhealth or Mobile application* or mobile technolog* or mobile healthcare or smartphone* or e-Health or m-Health or tele-Health or mobile device* or smart-phone or cell-phone or tele-rehabilitation or computer-based intervention* or Internet or Web or Internet-based or Web-based or computer-based or Online intervention* or Web based intervention* or Internet based intervention* or Videoconferenc* or Mobile phone* or Smart phone* or Cell phone* or Telephone* or Tablet* or App-based or App* or Text messag* or Texting* or iCBT or Internet-based cognitive behavio?r or computer-based cognitive behavio?r or (Wearable adj3 technolog*) or (Wearable adj3 device*) or (Electronic adj3 device*) or sports watch* or smartwatch* or activity tracker* or apple watch* or Activity tracker* or Fitness tracker*).mp. |
| 6 | 4 or 5 |
| 7 | exercise/ or cool-down exercise/ or gymnastics/ or muscle stretching exercises/ or exp Physical Fitness/ or exp Physical Conditioning, Human/ or Warm-Up Exercise/ or physical endurance/ or exercise tolerance/ or exp Exercise Therapy/ or exp Sports/ or Sports Medicine/ or exp Exercise Movement Techniques/ or exp Walking/ or exp Running/ or Swimming/ or Diving/ or "Physical Education and Training"/ or tai ji/ or yoga/ or Dance Therapy/ or Leisure Activities/ |
| 8 | (Exercise* or aerobic or Physical activit* or Physical inactivit* or Calisthenic* or Gymnastic* or Stretching* or Physical fitness or Human physical training or Human physical conditioning or Physical endurance or endurance training or Physical stamina or Athletic* or Sport* or biking or cycling or bicycling or Pilates or Pilates-based or Ambulation or Walk* or Treadmill* or Active transport* or active living or Run or Running* or Jog or Jogging* or Swim* or Diving* or Physical education or Yoga or Tai chi or Tai ji or Tai-chi or Dance* or Dancing or Weight lifting* or weight training or Leisure activit* or gardening or Recreational activit* or recreation activit* or Strength training* or Resistance training* or Weight-bearing or Physical therap*).mp. |
| 9 | 7 or 8 |
| 10 | (Intervention* or program* or trial* or rct or quasi-experimental or randomi?ed controlled trial* or randomi?ed trial* or controlled clinical trial* or clinical trial* or randomi?ed clinical trial* or randomi?ed control trial* or wait-list or experiment*).mp. |
| 11 | 3 and 6 |
| 12 | 11 and 9 |
| 13 | 12 and 10 |

Search strategy for PsycInfo (EBSCOhost)

| Search number | Query | Filters |
| --- | --- | --- |
| S1 | (DE "Telemedicine" OR DE "Online Therapy" OR DE "Teleconferencing" OR DE "Teleconsultation" OR DE "Telerehabilitation" OR DE "Computer Assisted Therapy" OR DE "Mobile Applications" OR DE "Electronic Health Services" OR DE "Digital Interventions" OR DE "Mobile Health" OR DE "Precision Medicine" OR DE "Wearable Devices" OR DE "Mobile Devices" OR DE "Mobile Phones" OR DE "Tablet Computers" OR DE "Smartphones" ) |  |
| S2 | “Mobile health” or Telehealth or Telemedicine or Telerehabilitation or E-rehab* or Ehealth or Mhealth or “Mobile application*” or “mobile technolog*” or “mobile healthcare” or smartphone* or e-Health or m-Health or tele-Health or “mobile device*” or smart-phone or cell-phone or tele-rehabilitation or “computer-based intervention*” or Internet or Web or Internet-based or Web-based or computer-based or “Online intervention*” or “Web based intervention*” or “Internet based intervention*” or Videoconferenc* or “Mobile phone*” or “Smart phone*” or “Cell phone*” or “Telephone*” or Tablet* or App-based or App* or “Text messag*” or Texting* or iCBT or “Internet-based cognitive behavio#r” or “computer-based cognitive behavio#r” or (Wearable N3 technolog*) or (Wearable N3 device*) or (Electronic N3 device*) or “sports watch*” or smartwatch* or “activity tracker*” or “apple watch*” or “Activity tracker*” or “Fitness tracker*” |  |
| S3 | S1 OR S2 |  |
| S4 | (DE "Physical Activity" OR DE "Actigraphy" OR DE "Exercise" OR DE "Physical Fitness" OR DE "Aerobic Exercise" OR DE "Weightlifting" OR DE "Yoga" OR DE "Activity Level" OR DE "Health Behavior") |  |
| S5 | Exercise* or aerobic or “Physical activit*” or “Physical inactivit*” or Calisthenic* or Gymnastic* or Stretching* or “Physical fitness” or “Human physical training” or “Human physical conditioning” or “Physical endurance” or “endurance training” or “Physical stamina” or Athletic* or Sport* or biking or cycling or bicycling or Pilates or Pilates-based or Ambulation or Walk* or Treadmill* or “Active transport*” or “active living” or Run or Running* or Jog or Jogging* or Swim* or Diving* or “Physical education” or Yoga or “Tai chi” or “Tai ji” or Tai-chi or Dance* or Dancing or “Weight lifting*” or “weight training” or “Leisure activit*” or gardening or “Recreational activit*” or “recreation activit*” or “Strength training*” or “Resistance training*” or Weight-bearing or “Physical therap*” |  |
| S6 | S4 OR S5 |  |
| S7 | (DE "Major Depression" OR DE "Anaclitic Depression" OR DE "Dysthymic Disorder" OR DE "Endogenous Depression" OR DE "Late Life Depression" OR DE "Postpartum Depression" OR DE "Reactive Depression" OR DE "Recurrent Depression" OR DE "Treatment Resistant Depression" ) |  |
| S8 | Depress* or melancholia* or Dysthymi* or “Mood disorder*” or “affective disorder*” or “Affective symptom*” or “Adjustment disorder*” or “Reactive disorder*” |  |
| S9 | S7 OR S8 |  |
| S10 | (TI Intervention* OR program* OR trial* OR rct OR "quasi experimental" OR quasi-experimental OR "randomi#ed controlled trial*" OR "randomi#ed trial*" OR “controlled clinical trial*” OR “clinical trial*” OR “randomi#ed clinical trial*” OR “randomi#ed control trial*” OR wait-list OR experiment*) |  |
| S11 | (AB Intervention* OR program* OR trial* OR rct OR "quasi experimental" OR quasi-experimental OR "randomi#ed controlled trial*" OR "randomi#ed trial*" OR “controlled clinical trial*” OR “clinical trial*” OR “randomi#ed clinical trial*” OR “randomi#ed control trial*” OR wait-list OR experiment*) |  |
| S12 | S10 OR S11 |  |
| S13 | S3 AND S6 |  |
| S14 | S9 AND S13 |  |
| S15 | S12 AND S14 |  |

Search strategy for EMBASE(via Ovid)

| Search number | Query |
| --- | --- |
| 1 | depression/ or Mood Disorder/ or Affective Symptoms/ or Adjustment Disorders/ |
| 2 | (Depress* or melancholia* or Dysthymi* or Mood disorder* or affective disorder* or Affective symptom* or Adjustment disorder* or Reactive disorder*).mp. [mp=title, abstract, heading word, drug trade name, original title, device manufacturer, drug manufacturer, device trade name, keyword, floating subheading word, candidate term word] |
| 3 | 1 or 2 |
| 4 | exp telehealth/ or mobile health application/ or web-based intervention/ or videoconferencing/ or wearable computer/ or actigraph/ or exp smart watch/ or exp sports equipment/ |
| 5 | (Mobile health or Telehealth or Telemedicine or Telerehabilitation or E-rehab* or Ehealth or Mhealth or Mobile application* or mobile technolog* or mobile healthcare or smartphone* or e-Health or m-Health or tele-Health or mobile device* or smart-phone or cell-phone or tele-rehabilitation or computer-based intervention* or Internet or Web or Internet-based or Web-based or computer-based or Online intervention* or Web based intervention* or Internet based intervention* or Videoconferenc* or Mobile phone* or Smart phone* or Cell phone* or Telephone* or Tablet* or App-based or App* or Text messag* or Texting* or iCBT or Internet-based cognitive behavio?r or computer-based cognitive behavio?r or (Wearable adj3 technolog*) or (Wearable adj3 device*) or (Electronic adj3 device*) or sports watch* or smartwatch* or activity tracker* or apple watch* or Activity tracker* or Fitness tracker*).mp. |
| 6 | 4 or 5 |
| 7 | exp physical activity/ or exp kinesiotherapy/ or exp exercise/ or exp sport/ or fitness/ or physical education/ or sports medicine/ or dance therapy/ or endurance/ |
| 8 | (Exercise* or aerobic or Physical activit* or Physical inactivit* or Calisthenic* or Gymnastic* or Stretching* or Physical fitness or Human physical training or Human physical conditioning or Physical endurance or endurance training or Physical stamina or Athletic* or Sport* or biking or cycling or bicycling or Pilates or Pilates-based or Ambulation or Walk* or Treadmill* or Active transport* or active living or Run or Running* or Jog or Jogging* or Swim* or Diving* or Physical education or Yoga or Tai chi or Tai ji or Tai-chi or Dance* or Dancing or Weight lifting* or weight training or Leisure activit* or gardening or Recreational activit* or recreation activit* or Strength training* or Resistance training* or Weight-bearing or Physical therap*).mp. |
| 9 | 7 or 8 |
| 10 | 3 and 6 |
| 11 | 9 and 10 |
| 12 | (Intervention* or program* or trial* or rct or quasi-experimental or randomi?ed controlled trial* or randomi?ed trial* or controlled clinical trial* or clinical trial* or randomi?ed clinical trial* or randomi?ed control trial* or wait-list or experiment*).mp. |
| 13 | 11 and 12 |

Search strategy for Web of Science

| Search number | Query | Filters |
| --- | --- | --- |
| #1 | TS=(depress* or dysthymi* or distress* or “common mental health” or “mood disorder*” or “affective disorder*” or “affective symptom*” or “adjustment disorder*”) |  |
| #2 | TS=(“Mobile application*” or “mobile technolog*” or “mobile healthcare” or mHealth or eHealth or teleHealth or digital or internet-based or web-based or app-based or apps or “mobile phone*” or smartphone* or computer* or tablet* or virtual or online or video* or e-Health or m-Health or tele-Health or “mobile health” or “mobile device*” or smart-phone or “smart phone*” or “cell phone*” or cell-phone or telerehabilitation or tele-rehabilitation or “computer-based intervention*” or “wearable technolog*” or e-rehab* or iCBT or “computer-based cognitive behavio$r” or computer-based or “sports watch*” or smartwatch* or “activity tracker*” or “apple watch*” or “text messag*”) |  |
| #3 | TS=(“Physical activit*” or “Physical inactivit*” or “Sedentary behavio*” or aerobic or “physical fitness” or walk* or run or running or yoga or “strength training” or sports or “active living” or “active transport” or “leisure activit*” or “resistance training” or “endurance training” or “weight training” or Tai-chi or swim* or jog or jogging or dance* or dancing or gardening or treadmill* or biking or cycling or pilates or “recreation activit*” or “physical therap*”) |  |
| #4 | AB=(Intervention* OR program* OR trial* OR rct OR quasi-experimental OR "randomi$ed controlled trial*" OR "randomi$ed trial*" OR “controlled clinical trial*” OR “clinical trial*” OR “randomi$ed clinical trial*” OR “randomi$ed control trial*” OR wait-list OR experiment*) |  |
| #5 | AB=(allocat* OR control) |  |
| #6 | #4 OR #5 |  |
| #7 | #1 AND #2 AND #3 AND #6 |  |

Search strategy for SportDiscus (EBSCOhost)

| Search number | Query | Thesaurus |
| --- | --- | --- |
| S1 | TX "Mobile application*" OR "mobile technolog*" OR "mobile healthcare" OR mHealth OR eHealth OR teleHealth OR digital OR internet-based OR web-based OR app-based OR apps OR "mobile phone*" OR smartphone* OR computer* OR tablet* OR virtual OR online OR video* OR e-Health OR m-Health OR tele-Health OR "mobile health" OR "mobile device*" OR smart-phone OR "smart phone*" OR "cell phone*" OR cell-phone OR telerehabilitation OR “tele-rehabilitation” OR “computer-based intervention*” OR “wearable technolog*” OR e-rehab* OR iCBT OR “computer-based cognitive behavio#r” OR computer-based OR “sports watch*” OR smartwatch* OR “activity tracker*” OR “apple watch*” OR “text messag*” |  |
| S2 | (DE "EXERCISE" ) OR (DE "PHYSICAL activity") | Explode “exercise” |
| S3 | TX "Physical activit*" OR "Physical inactivit*" OR "Sedentary behavio*" OR aerobic OR "physical fitness" OR walk* OR run OR running OR yoga OR "strength training" OR sports OR "active living" OR "active transport" OR "leisure activit*" OR "resistance training" OR "endurance training" OR "weight training" OR Tai-chi OR swim* OR jog OR jogging OR dance* OR dancing OR gardening OR treadmill* OR biking OR cycling OR pilates OR “recreation activit*” OR “physical therap*” |  |
| S4 | S2 OR S3 |  |
| S5 | (DE "MENTAL depression" OR DE "DEPRESSION in college students" ) | Explode “mental depression” |
| S7 | TX (depress* OR dysthymi* OR distress* OR “common mental health” OR “mood disorder*” OR “affective disorder*” OR “affective symptom*” OR “adjustment disorder*”) |  |
| S7 | S1 AND S4 AND S5 |  |
| S8 | (AB Intervention* OR program* OR trial* OR rct OR "quasi experimental" OR quasi-experimental OR "randomi#ed controlled trial*" OR "randomi#ed trial*" OR “controlled clinical trial*” OR “clinical trial*” OR “randomi#ed clinical trial*” OR “randomi#ed control trial*” OR wait-list OR experiment* OR allocat* OR control) |  |
| S9 | (TI Intervention* OR program* OR trial* OR rct OR "quasi experimental" OR quasi-experimental OR "randomi#ed controlled trial*" OR "randomi#ed trial*" OR “controlled clinical trial*” OR “clinical trial*” OR “randomi#ed clinical trial*” OR “randomi#ed control trial*” OR wait-list OR experiment* OR allocat* OR control) |  |
| S10 | S9 OR S9 |  |
| S1 | S6 AND S9 |  |

Search strategy in CINAHL (EBSCOhost)

| Search number | Query |
| --- | --- |
| S1 | (MH "Telemedicine+") OR (MH "Telehealth") OR (MH "Videoconferencing") OR (MH "Mobile Applications") OR (MH "Smartphone") OR (MH "Internet-Based Intervention") OR (MH "Accelerometers") |
| S2 | TX “Mobile health” or Telehealth or Telemedicine or Telerehabilitation or E-rehab* or Ehealth or Mhealth or “Mobile application*” or “mobile technolog*” or “mobile healthcare” or smartphone* or e-Health or m-Health or tele-Health or “mobile device*” or smart-phone or cell-phone or tele-rehabilitation or “computer-based intervention*” or Internet or Web or Internet-based or Web-based or computer-based or “Online intervention*” or “Web based intervention*” or “Internet based intervention*” or Videoconferenc* or “Mobile phone*” or “Smart phone*” or “Cell phone*” or “Telephone*” or Tablet* or App-based or App* or “Text messag*” or Texting* or iCBT or “Internet-based cognitive behavio#r” or “computer-based cognitive behavio#r” or (Wearable N3 technolog*) or (Wearable N3 device*) or (Electronic N3 device*) or “sports watch*” or smartwatch* or “activity tracker*” or “apple watch*” or “Activity tracker*” or “Fitness tracker*” |
| S3 | S1 OR S2 |
| S4 | (MH "Exercise+") OR (MH "Activities of Daily Living") OR (MH "Aerobic Exercises+") OR (MH "Muscle Strengthening+") OR (MH "Walking+") OR (MH "Upper Extremity Exercises+") OR (MH "Physical Activity") OR (MH "Physical Fitness+") OR (MH "Leisure Activities") OR (MH "Sports+") OR (MH "Resistance Training") OR (MH "Dance Therapy") OR (MH "Physical Therapy+") OR (MH "Telerehabilitation") OR (MH "Home Physical Therapy") OR (MH "Rehabilitation, Athletic") OR (MH "Weight Lifting") |
| S5 | TX Exercise* or aerobic or “Physical activit*” or “Physical inactivit*” or Calisthenic* or Gymnastic* or Stretching* or “Physical fitness” or “Human physical training” or “Human physical conditioning” or “Physical endurance” or “endurance training” or “Physical stamina” or Athletic* or Sport* or biking or cycling or bicycling or Pilates or Pilates-based or Ambulation or Walk* or Treadmill* or “Active transport*” or “active living” or Run or Running* or Jog or Jogging* or Swim* or Diving* or “Physical education” or Yoga or “Tai chi” or “Tai ji” or Tai-chi or Dance* or Dancing or “Weight lifting*” or “weight training” or “Leisure activit*” or gardening or “Recreational activit*” or “recreation activit*” or “Strength training*” or “Resistance training*” or Weight-bearing or “Physical therap*” |
| S6 | S5 OR S6 |
| S7 | (MH "Depression+") OR (MH "Adjustment Disorders+") OR (MH "Affective Disorders, Psychotic") |
| S8 | TX (Depress* or melancholia* or Dysthymi* or “Mood disorder*” or “affective disorder*” or “Affective symptom*” or “Adjustment disorder*” or “Reactive disorder*”) |
| S9 | S7 OR S8 |
| S10 | S3 AND S6 AND S9 |
| S11 | (AB Intervention* OR program* OR trial* OR rct OR "quasi experimental" OR quasi-experimental OR "randomi#ed controlled trial*" OR "randomi#ed trial*" OR “controlled clinical trial*” OR “clinical trial*” OR “randomi#ed clinical trial*” OR “randomi#ed control trial*” OR wait-list OR experiment*) |
| S12 | (TI Intervention* OR program* OR trial* OR rct OR "quasi experimental" OR quasi-experimental OR "randomi#ed controlled trial*" OR "randomi#ed trial*" OR “controlled clinical trial*” OR “clinical trial*” OR “randomi#ed clinical trial*” OR “randomi#ed control trial*” OR wait-list OR experiment*) |
| S13 | S11 OR S12 |
| S14 | S10 AND S13 |

Search strategy for the Cochrane Central Register of Controlled Trials (CENTRAL)(via Ovid)

| Search number | Query |
| --- | --- |
| 1 | exp depression/ or exp depressive disorder/ |
| 2 | (depress* or dysthymi* or distress* or common mental health or mood disorder* or affective disorder* or affective symptom* or adjustment disorder*).mp. [mp=title, abstract, heading word, drug trade name, original title, device manufacturer, drug manufacturer, device trade name, keyword, floating subheading word, candidate term word] |
| 3 | 1 or 2 |
| 4 | exp telemedicine/ |
| 5 | (Mobile application* or mobile technolog* or mobile healthcare or mHealth or eHealth or teleHealth or digital or internet-based or web-based or app-based or apps or mobile phone* or smartphone* or computer* or tablet* or virtual or online or video* or e-Health or m-Health or tele-Health or mobile health or mobile device* or smart-phone or smart phone* or cell phone* or cell-phone or telerehabilitation or tele-rehabilitation or computer-based intervention* or wearable technolog* or e-rehab* or iCBT or computer-based cognitive behavio?r or computer-based or sports watch* or smartwatch* or activity tracker* or apple watch* or text messag*).mp. |
| 6 | 4 or 5 |
| 7 | exp exercise/ |
| 8 | (Physical activit* or Physical inactivit* or Sedentary behavio* or aerobic or physical fitness or walk* or run or running or yoga or strength training or sports or active living or active transport or leisure activit* or resistance training or endurance training or weight training or Tai-chi or swim* or jog or jogging or dance* or dancing or gardening or treadmill* or biking or cycling or pilates or recreation activit* or physical therap*).mp. |
| 9 | 7 or 8 |
| 10 | 3 and 6 and 9 |

Search strategy for OpenGrey

(depress* OR dysthymi* OR distress* OR “common mental health” OR “mood disorder*” OR “affective disorder*” OR “affective symptom*” OR “adjustment disorder*”) AND ("physical activity" OR “physical inactivity” OR “sedentary behavio*” OR "exercise" OR "aerobic" OR "run" OR "running" OR "yoga" OR "jog" OR "jogging" OR "swim" OR "swimming" OR "physical fitness" OR "active transport" OR "leisure activit*" OR “endurance training” OR “resistance training” OR "weight training" OR "Tai Chi" OR "sports" OR "walk" OR "walking") AND ("eHealth" OR "mHealth" OR "telemedicine" OR "app" OR "mobile" OR "online" OR "mobile application" OR "telehealth")

Search strategy for ProQuest

| Search number | Query | Filters |
| --- | --- | --- |
| S1 | noft(Intervention* or program* or trial* or rct or quasi-experimental or randomized controlled trial* or randomized trial* or controlled clinical trial* or clinical trial* or randomized clinical trial* or randomized control trial* or wait-list or experiment* or allocat* or control) | Limit to full text |
| S2 | noft(depress* or dysthymi* or distress* or common mental health or mood disorder* or affective disorder* or affective symptom* or adjustment disorder*) | Limit to full text |
| S3 | noft(Mobile application* or mobile technolog* or mobile healthcare or mHealth or eHealth or teleHealth or digital or internet-based or web-based or app-based or apps or mobile phone* or smartphone* or computer* or tablet* or virtual or online or video* or e-Health or m-Health or tele-Health or mobile health or mobile device* or smart-phone or smart phone* or cell phone* or cell-phone or telerehabilitation or tele-rehabilitation or computer-based intervention* or wearable technolog* or e-rehab* or iCBT or computer-based cognitive behavio?r or computer-based or sports watch* or smartwatch* or activity tracker* or apple watch* or text messag*) | Limit to full text |
| S4 | noft(Physical activit* or Physical inactivit* or Sedentary behavio* or aerobic or physical fitness or walk* or run or running or yoga or strength training or sports or active living or active transport or leisure activit* or resistance training or endurance training or weight training or Tai-chi or swim* or jog or jogging or dance* or dancing or gardening or treadmill* or biking or cycling or pilates or recreation activit* or physical therap*) | Limit to full text |
| S5 | 1 AND 2 AND 3 AND 4 |  |
