# Supplementary material for: The Effectiveness of Internet-Guided Self-help Interventions to Promote Physical Activity Among Individuals With Depression: Systematic Review
Source: JMIR Ment Health. 2022 Dec 12;9(12):e38049. doi: 10.2196/38049 (PMC9793299; doi:10.2196/38049)
Supplement: Multimedia Appendix 3 [file mental_v9i12e38049_app3.docx]

Multimedia Appendix 3. Search strategies for the second round of searches

Search Strategy for MEDLINE (Ovid)

| Search number | Query |
| --- | --- |
| 1 | depression/ or exp Depressive Disorder/ or Dysthymic Disorder/ or Mood Disorders/ or Affective Symptoms/ or Adjustment Disorders/ |
| 2 | Depress*.mp. [mp=title, abstract, heading word, drug trade name, original title, device manufacturer, drug manufacturer, device trade name, keyword, floating subheading word, candidate term word] |
| 3 | 1 or 2 |
| 4 | exp Internet/ or exp Cell Phone/ |
| 5 | (Mobile health or E-rehab* or Ehealth or Mhealth or Mobile application* or mobile technolog* or mobile healthcare or smartphone* or e-Health or m-Health or mobile device* or smart-phone or cell-phone or computer-based intervention* or Internet or Web or computer or Internet-based or Web-based or computer-based or Online intervention* or Web based intervention* or Internet based intervention* or Mobile phone* or Smart phone* or Cell phone* or App-based or App*).mp. |
| 6 | 4 or 5 |
| 7 | exercise/ or cool-down exercise/ or gymnastics/ or muscle stretching exercises/ or exp Physical Fitness/ or exp Physical Conditioning, Human/ or Warm-Up Exercise/ or physical endurance/ or exercise tolerance/ or exp Exercise Therapy/ or exp Sports/ or Sports Medicine/ or exp Exercise Movement Techniques/ or exp Walking/ or exp Running/ or Swimming/ or Diving/ or "Physical Education and Training"/ or tai ji/ or yoga/ or Dance Therapy/ or Leisure Activities/ |
| 8 | (Exercise* or aerobic or Physical activit* or Physical inactivit* or Stretching* or Physical fitness or Physical endurance or endurance training or Physical stamina or Athletic* or Sport* or biking or cycling or bicycling or Pilates or Pilates-based or Ambulation or Walk* or Treadmill* or Active transport* or active living or Run or Running* or Jog or Jogging* or Swim* or Diving* or Physical education or Yoga or Tai chi or Tai ji or Tai-chi or Dance* or Dancing or Weight lifting* or weight training or Leisure activit* or gardening or Recreational activit* or recreation activit* or Strength training* or Resistance training* or Weight-bearing or Physical therap*).mp. |
| 9 | 7 or 8 |
| 10 | (rct or quasi-experimental or randomi?ed controlled trial* or randomi?ed trial* or randomi?ed clinical trial* or randomi?ed control trial* or wait-list or experiment* or randomly).mp. |
| 11 | 3 and 6 |
| 12 | 11 and 9 |
| 13 | 12 and 10 |

Search strategy for EMBASE(via Ovid)

| Search number | Query |
| --- | --- |
| 1 | depression/ or Mood Disorder/ or Affective Symptoms/ or Adjustment Disorders/ |
| 2 | Depress*.mp. [mp=title, abstract, heading word, drug trade name, original title, device manufacturer, drug manufacturer, device trade name, keyword, floating subheading word, candidate term word] |
| 3 | 1 or 2 |
| 4 | mobile health application/ or web-based intervention/ |
| 5 | (Mobile health or E-rehab* or Ehealth or Mhealth or Mobile application* or mobile technolog* or mobile healthcare or smartphone* or e-Health or m-Health or mobile device* or smart-phone or cell-phone or computer-based intervention* or Internet or Web or computer or Internet-based or Web-based or computer-based or Online intervention* or Web based intervention* or Internet based intervention* or Mobile phone* or Smart phone* or Cell phone* or App-based or App*).mp. |
| 6 | 4 or 5 |
| 7 | exp physical activity/ or exp kinesiotherapy/ or exp exercise/ or exp sport/ or fitness/ or physical education/ or sports medicine/ or dance therapy/ or endurance/ |
| 8 | (Exercise* or aerobic or Physical activit* or Physical inactivit* or Stretching* or Physical fitness or Physical endurance or endurance training or Physical stamina or Athletic* or Sport* or biking or cycling or bicycling or Pilates or Pilates-based or Ambulation or Walk* or Treadmill* or Active transport* or active living or Run or Running* or Jog or Jogging* or Swim* or Diving* or Physical education or Yoga or Tai chi or Tai ji or Tai-chi or Dance* or Dancing or Weight lifting* or weight training or Leisure activit* or gardening or Recreational activit* or recreation activit* or Strength training* or Resistance training* or Weight-bearing or Physical therap*).mp. |
| 9 | 7 or 8 |
| 10 | 3 and 6 |
| 11 | 9 and 10 |
| 12 | (rct or quasi-experimental or randomi?ed controlled trial* or randomi?ed trial* or randomi?ed clinical trial* or randomi?ed control trial* or wait-list or experiment* or randomly).mp. |
| 13 | 11 and 12 |

Search strategy for the Cochrane Central Register of Controlled Trials (CENTRAL)(via Ovid)

| Search number | Query |
| --- | --- |
| 1 | Depress*.mp. [mp=title, abstract, heading word, drug trade name, original title, device manufacturer, drug manufacturer, device trade name, keyword, floating subheading word, candidate term word] |
| 2 | (Mobile health or E-rehab* or Ehealth or Mhealth or Mobile application* or mobile technolog* or mobile healthcare or smartphone* or e-Health or m-Health or mobile device* or smart-phone or cell-phone or computer-based intervention* or Internet or Web or computer or Internet-based or Web-based or computer-based or Online intervention* or Web based intervention* or Internet based intervention* or Mobile phone* or Smart phone* or Cell phone* or App-based or App*).mp. |
| 3 | exp exercise/ or exp physical endurance/ or physical fitness/ or exp sports/ or exp exercise therapy/ or Dance Therapy/ or Sports Medicine/ |
| 4 | (Exercise* or aerobic or Physical activit* or Physical inactivit* or Stretching* or Physical fitness or Physical endurance or endurance training or Physical stamina or Athletic* or Sport* or biking or cycling or bicycling or Pilates or Pilates-based or Ambulation or Walk* or Treadmill* or Active transport* or active living or Run or Running* or Jog or Jogging* or Swim* or Diving* or Physical education or Yoga or Tai chi or Tai ji or Tai-chi or Dance* or Dancing or Weight lifting* or weight training or Leisure activit* or gardening or Recreational activit* or recreation activit* or Strength training* or Resistance training* or Weight-bearing or Physical therap*).mp. |
| 5 | 3 or 4 |
| 6 | 1 and 2 |
| 7 | 6 and 5 |

Search strategy for PsycInfo (EBSCOhost)

| Search number | Query | Filters |
| --- | --- | --- |
| S1 | (DE "Online Therapy" OR DE "Computer Assisted Therapy" OR DE "Mobile Applications" OR DE "Electronic Health Services" OR DE "Digital Interventions" OR DE "Mobile Health" OR DE "Mobile Devices" OR DE "Mobile Phones" OR DE "Tablet Computers" OR DE "Smartphones" ) |  |
| S2 | “Mobile health” or E-rehab* or Ehealth or Mhealth or “Mobile application*” or “mobile technolog*” or “mobile healthcare” or smartphone* or e-Health or m-Health or “mobile device*” or smart-phone or cell-phone or “computer-based intervention*” or Internet or Web or computer or Internet-based or Web-based or computer-based or “Online intervention*” or “Web based intervention*” or “Internet based intervention*” or “Mobile phone*” or “Smart phone*” or “Cell phone*” or App-based or App* |  |
| S3 | S1 OR S2 |  |
| S4 | (DE "Physical Activity" OR DE "Exercise" OR DE "Physical Fitness" OR DE "Aerobic Exercise" OR DE "Weightlifting" OR DE "Yoga") |  |
| S5 | Exercise* or aerobic or “Physical activit*” or “Physical inactivit*” or Stretching* or “Physical fitness” or “Human physical training” or “Human physical conditioning” or “Physical endurance” or “endurance training” or “Physical stamina” or Athletic* or Sport* or biking or cycling or bicycling or Pilates or Pilates-based or Ambulation or Walk* or Treadmill* or “Active transport*” or “active living” or Run or Running* or Jog or Jogging* or Swim* or Diving* or “Physical education” or Yoga or “Tai chi” or “Tai ji” or Tai-chi or Dance* or Dancing or “Weight lifting*” or “weight training” or “Leisure activit*” or gardening or “Recreational activit*” or “recreation activit*” or “Strength training*” or “Resistance training*” or Weight-bearing or “Physical therap*” |  |
| S6 | S4 OR S5 |  |
| S7 | (DE "Major Depression" OR DE "Anaclitic Depression" OR DE "Dysthymic Disorder" OR DE "Endogenous Depression" OR DE "Late Life Depression" OR DE "Postpartum Depression" OR DE "Reactive Depression" OR DE "Recurrent Depression" OR DE "Treatment Resistant Depression" ) |  |
| S8 | Depress* |  |
| S9 | S7 OR S8 |  |
| S10 | (TI rct OR "quasi experimental" OR quasi-experimental OR "randomi#ed controlled trial*" OR "randomi#ed trial*" OR “randomi#ed clinical trial*” OR “randomi#ed control trial*” OR wait-list OR experiment*) |  |
| S11 | (AB rct OR "quasi experimental" OR quasi-experimental OR "randomi#ed controlled trial*" OR "randomi#ed trial*" OR “randomi#ed clinical trial*” OR “randomi#ed control trial*” OR wait-list OR experiment* OR randomly) |  |
| S12 | S10 OR S11 |  |
| S13 | S3 AND S6 |  |
| S14 | S9 AND S13 |  |
| S15 | S12 AND S14 |  |

Search strategy for Web of Science

| Search number | Query |
| --- | --- |
| #1 | TS=Depress* |
| #2 | TS=(“Mobile health” or E-rehab* or Ehealth or Mhealth or “Mobile application*” or “mobile technolog*” or “mobile healthcare” or smartphone* or e-Health or m-Health or “mobile device*” or smart-phone or cell-phone or “computer-based intervention*” or Internet or Web or computer or Internet-based or Web-based or computer-based or “Online intervention*” or “Web based intervention*” or “Internet based intervention*” or “Mobile phone*” or “Smart phone*” or “Cell phone*” or Tablet* or App-based or App*) |
| #3 | TS=(Exercise* or aerobic or “Physical activit*” or “Physical inactivit*” or Stretching* or “Physical fitness” or “Human physical training” or “Human physical conditioning” or “Physical endurance” or “endurance training” or “Physical stamina” or Athletic* or Sport* or biking or cycling or bicycling or Pilates or Pilates-based or Ambulation or Walk* or Treadmill* or “Active transport*” or “active living” or Run or Running* or Jog or Jogging* or Swim* or Diving* or “Physical education” or Yoga or “Tai chi” or “Tai ji” or Tai-chi or Dance* or Dancing or “Weight lifting*” or “weight training” or “Leisure activit*” or gardening or “Recreational activit*” or “recreation activit*” or “Strength training*” or “Resistance training*” or Weight-bearing or “Physical therap*”) |
| #4 | TS=(rct OR quasi-experimental OR "randomi$ed controlled trial*" OR "randomi$ed trial*" OR “randomi$ed clinical trial*” OR “randomi$ed control trial*” OR wait-list OR experiment* OR randomly) |
| #5 | #1 AND #2 AND #3AND #4 |

Search strategy for SportDiscus (EBSCOhost)

| Search number | Query | Thesaurus |
| --- | --- | --- |
| S1 | “Mobile health” or E-rehab* or Ehealth or Mhealth or “Mobile application*” or “mobile technolog*” or “mobile healthcare” or smartphone* or e-Health or m-Health or tele-Health or “mobile device*” or smart-phone or cell-phone or “computer-based intervention*” or Internet or Web or computer or Internet-based or Web-based or computer-based or “Online intervention*” or “Web based intervention*” or “Internet based intervention*” or “Mobile phone*” or “Smart phone*” or “Cell phone*” or Tablet* or App-based or App* |  |
| S2 | DE "EXERCISE" OR DE "EXERCISE videos" OR DE "PHYSICAL activity" OR DE "PHYSICAL education" OR DE "PHYSICAL fitness" OR DE "PHYSICAL training & conditioning" OR DE "SPORTS" OR DE "WARMUP" | Explode “exercise” |
| S3 | Exercise* or aerobic or “Physical activit*” or “Physical inactivit*” or Stretching* or “Physical fitness” or “Human physical training” or “Human physical conditioning” or “Physical endurance” or “endurance training” or “Physical stamina” or Athletic* or Sport* or biking or cycling or bicycling or Pilates or Pilates-based or Ambulation or Walk* or Treadmill* or “Active transport*” or “active living” or Run or Running* or Jog or Jogging* or Swim* or Diving* or “Physical education” or Yoga or “Tai chi” or “Tai ji” or Tai-chi or Dance* or Dancing or “Weight lifting*” or “weight training” or “Leisure activit*” or gardening or “Recreational activit*” or “recreation activit*” or “Strength training*” or “Resistance training*” or Weight-bearing or “Physical therap*” |  |
| S4 | S2 OR S3 |  |
| S5 | (DE "MENTAL depression" OR DE "DEPRESSION in college students" OR DE "MOOD (Psychology)" OR DE "AFFECTIVE disorders") | Explode “mental depression” |
| S6 | Depress* |  |
| S7 | S5 OR S6 |  |
| S8 | S1 AND S4 AND S7 |  |
| S9 | (AB rct OR "quasi experimental" OR quasi-experimental OR "randomi#ed controlled trial*" OR "randomi#ed trial*" OR “randomi#ed clinical trial*” OR “randomi#ed control trial*” OR wait-list OR experiment* or randomly) |  |
| S10 | (TI rct OR "quasi experimental" OR quasi-experimental OR "randomi#ed controlled trial*" OR "randomi#ed trial*" OR “randomi#ed clinical trial*” OR “randomi#ed control trial*” OR wait-list OR experiment*) |  |
| S11 | S9 OR S10 |  |
| S12 | S8 AND S11 |  |

Search strategy in CINAHL (EBSCOhost)

| Search number | Query |
| --- | --- |
| S1 | (MH "Mobile Applications") OR (MH "Smartphone") OR (MH "Internet-Based Intervention") |
| S2 | AB “Mobile health” or E-rehab* or Ehealth or Mhealth or “Mobile application*” or “mobile technolog*” or “mobile healthcare” or smartphone* or e-Health or m-Health or “mobile device*” or smart-phone or cell-phone or “computer-based intervention*” or Internet or Web or Internet-based or Web-based or computer-based or “Online intervention*” or “Web based intervention*” or “Internet based intervention*” or “Mobile phone*” or “Smart phone*” or “Cell phone*” or Tablet* or App-based or App* |
| S3 | S1 OR S2 |
| S4 | (MH "Exercise+") OR (MH "Activities of Daily Living") OR (MH "Aerobic Exercises+") OR (MH "Muscle Strengthening+") OR (MH "Walking+") OR (MH "Physical Activity") OR (MH "Physical Fitness+") OR (MH "Leisure Activities") OR (MH "Sports+") OR (MH "Resistance Training") OR (MH "Dance Therapy") OR (MH "Physical Therapy+") OR (MH "Home Physical Therapy") OR (MH "Rehabilitation, Athletic") OR (MH "Weight Lifting") |
| S5 | AB Exercise* or aerobic or “Physical activit*” or “Physical inactivit*” or Stretching* or “Physical fitness” or “Human physical training” or “Human physical conditioning” or “Physical endurance” or “endurance training” or “Physical stamina” or Athletic* or Sport* or biking or cycling or bicycling or Pilates or Pilates-based or Ambulation or Walk* or Treadmill* or “Active transport*” or “active living” or Run or Running* or Jog or Jogging* or Swim* or Diving* or “Physical education” or Yoga or “Tai chi” or “Tai ji” or Tai-chi or Dance* or Dancing or “Weight lifting*” or “weight training” or “Leisure activit*” or gardening or “Recreational activit*” or “recreation activit*” or “Strength training*” or “Resistance training*” or Weight-bearing or “Physical therap*” |
| S6 | S5 OR S6 |
| S7 | (MH "Depression+") OR (MH "Adjustment Disorders+") OR (MH "Affective Disorders, Psychotic") |
| S8 | AB Depress* |
| S9 | S7 OR S8 |
| S10 | S3 AND S6 AND S9 |
| S11 | (AB rct OR "quasi experimental" OR quasi-experimental OR "randomi#ed controlled trial*" OR "randomi#ed trial*" OR “randomi#ed clinical trial*” OR “randomi#ed control trial*” OR wait-list OR experiment* OR randomly) |
| S12 | (TI rct OR "quasi experimental" OR quasi-experimental OR "randomi#ed controlled trial*" OR "randomi#ed trial*" OR “randomi#ed clinical trial*” OR “randomi#ed control trial*” OR wait-list OR experiment*) |
| S13 | S11 OR S12 |
| S14 | S10 AND S13 |

Search strategy for OpenGrey

((((Depress*) AND (Exercise* OR aerobic OR “Physical activit*” OR “Physical inactivit*”OR “Physical fitness” OR “Human physical training” OR “Human physical conditioning” OR “Physical endurance” OR “endurance training” OR “Physical stamina” OR Athletic* OR Sport* OR biking OR cycling OR bicycling OR Pilates OR Pilates-based OR Ambulation OR Walk* OR Treadmill* OR “Active transport*” OR “active living” OR Run OR Running* OR Jog OR Jogging* OR Swim* OR Diving* OR “Physical education” OR Yoga OR “Tai chi” OR “Tai ji” OR Tai-chi OR Dance* OR Dancing OR “Weight lifting*” OR “weight training” OR “Leisure activit*” OR gardening OR “Recreational activit*” OR “recreation activit*” OR “Strength training*” OR “Resistance training*” OR Weight-bearing OR “Physical therap*”)) AND (eHealth OR mHealth OR app* OR mobile OR online OR “mobile application” OR telehealth OR “mobile technolog*” OR “mobile healthcare” OR smartphone* OR e-Health OR m-Health OR Internet OR Web OR computer OR Internet-based OR Web-based OR computer-based OR “Online intervention*” OR “Web based intervention*”)) AND (rct OR “quasi experimental” OR quasi-experimental OR “randomized controlled trial*” OR “randomized trial*”OR “randomized clinical trial*” OR “randomized control trial*” OR wait-list OR experiment* OR randomly)) lang:"en"

Search strategy for ProQuest

| Search number | Query | Filters |
| --- | --- | --- |
| S1 | noft(rct or quasi-experimental or randomized controlled trial* or randomized trial* or randomized clinical trial* or randomized control trial* or wait-list or experiment* or randomly) | Limit to full text |
| S2 | noft(Depress* or melancholia* or Dysthymi* or Mood disorder* or affective disorder* or Affective symptom* or Adjustment disorder* or Reactive disorder*) | Limit to full text |
| S3 | noft(Mobile health or Telehealth or Telemedicine or Telerehabilitation or E-rehab* or Ehealth or Mhealth or Mobile application* or mobile technolog* or mobile healthcare or smartphone* or e-Health or m-Health or tele-Health or mobile device* or smart-phone or cell-phone or tele-rehabilitation or computer-based intervention* or Internet or Web or Internet-based or Web-based or computer-based or Online intervention* or Web based intervention* or Internet based intervention* or Videoconferenc* or Mobile phone* or Smart phone* or Cell phone* or Telephone* or Tablet* or App-based or App* or Text messag* or Texting* or iCBT or Internet-based cognitive behavior or computer-based cognitive behavior or (Wearable N/3 technolog*) or (Wearable N/3 device*) or (Electronic N/3 device*) or sports watch* or smartwatch* or activity tracker* or apple watch* or Activity tracker* or Fitness tracker*) | Limit to full text |
| S4 | noft(Exercise* or aerobic or Physical activit* or Physical inactivit* or Calisthenic* or Gymnastic* or Stretching* or Physical fitness or Human physical training or Human physical conditioning or Physical endurance or endurance training or Physical stamina or Athletic* or Sport* or biking or cycling or bicycling or Pilates or Pilates-based or Ambulation or Walk* or Treadmill* or Active transport* or active living or Run or Running* or Jog or Jogging* or Swim* or Diving* or Physical education or Yoga or Tai chi or Tai ji or Tai-chi or Dance* or Dancing or Weight lifting* or weight training or Leisure activit* or gardening or Recreational activit* or recreation activit* or Strength training* or Resistance training* or Weight-bearing or Physical therap*) | Limit to full text |
| S5 | 1 AND 2 AND 3 AND 4 |  |
